# Marine animal evolutionary developmental biology—Advances through technology development

**DOI:** 10.1111/eva.13456

**Published:** 2023-01-19

**Authors:** Katharina Stracke, Andreas Hejnol

**Affiliations:** ^1^ Department of Biological Sciences, Faculty of Mathematics and Natural Sciences University of Bergen Bergen Norway; ^2^ Institute of Systematic Zoology and Evolutionary Biology Friedrich‐Schiller‐University Jena Jena Germany

**Keywords:** development, evolution, marine invertebrates, phylogeny

## Abstract

Evolutionary developmental biology, the interdisciplinary effort of illuminating the conserved similarities and differences during animal development across all phylogenetic clades, has gained renewed interest in the past decades. As technology (immunohistochemistry, next‐generation sequencing, advanced imaging, and computational resources) has advanced, so has our ability of resolving fundamental hypotheses and overcoming the genotype–phenotype gap. This rapid progress, however, has also exposed gaps in the collective knowledge around the choice and representation of model organisms. It has become clear that evo‐devo requires a comparative, large‐scale approach including marine invertebrates to resolve some of the most urgent questions about the phylogenetic positioning and character traits of the last common ancestors. Many invertebrates at the base of the tree of life inhabit marine environments and have been used for some years due to their accessibility, husbandry, and morphology. Here, we briefly review the major concepts of evolutionary developmental biology and discuss the suitability of established model organisms to address current research questions, before focussing on the importance, application, and state‐of‐the‐art of marine evo‐devo. We highlight novel technical advances that progress evo‐devo as a whole.

## 1 INTRODUCTION

In 1866, Ernst Haeckel expressed the idea that the role of development is fundamental for understanding evolutionary theory through his “biogenetic law” that states “ontogeny recapitulates phylogeny,” meaning an organism passes through life forms resembling ancestral adult forms when developing from embryo to adult (Haeckel, 1866). After this early note of the connection between phylogeny and ontogeny nearly two centuries of research have passed, that demonstrated the connection is not as simple as Haeckel has imagined (Levit et al., 2022). Development itself is prone to evolutionary changes and this dissociates the by Haeckel assumed tight bound of development and evolution (Scholtz, 2005). Evolutionary developmental biologists therefore aim to understand the mechanisms that underly the evolutionary changes of developmental pathways and investigate how genomic changes lead to developmental changes which possibly lead to a change in the adult form among others. Behind the vast diversity of adult forms, we have a range of developmental pathways that lead to them. Examining the developmental biodiversity shows that each developmental stage can adapt to environmental changes which may or may not translate into the adult phenotype and possibly the whole life cycle (Garstang, 1922). This alone indicates that it is necessary to study developmental diversity to understand it better and to gain insights into the mechanisms that underly the broad changes during the developmental processes on the level of gene regulation, cell–cell interactions, and morphogenesis. Luckily, the recent decades were landmarked by amazing progress in the development of molecular, microscopic, and computational approaches. New technologies allow to study of developmental processes in more detail and with higher accuracy and are therefore driving the field forward. These technological advances not only allow to study development in more detail, but also allow to expand the range of organisms that can be studied. The last decades also led to a better understanding of how animal taxa are related to each other (Dunn et al., 2014), which ultimately provides the framework that is essential to understanding the time and direction of evolutionary changes on all biological levels (Hejnol & Lowe, 2015). Together, there has never been a better time to do comparative developmental biology of animals.

### 1.1 An expansion of research organisms is still necessary

**FIGURE 1:**
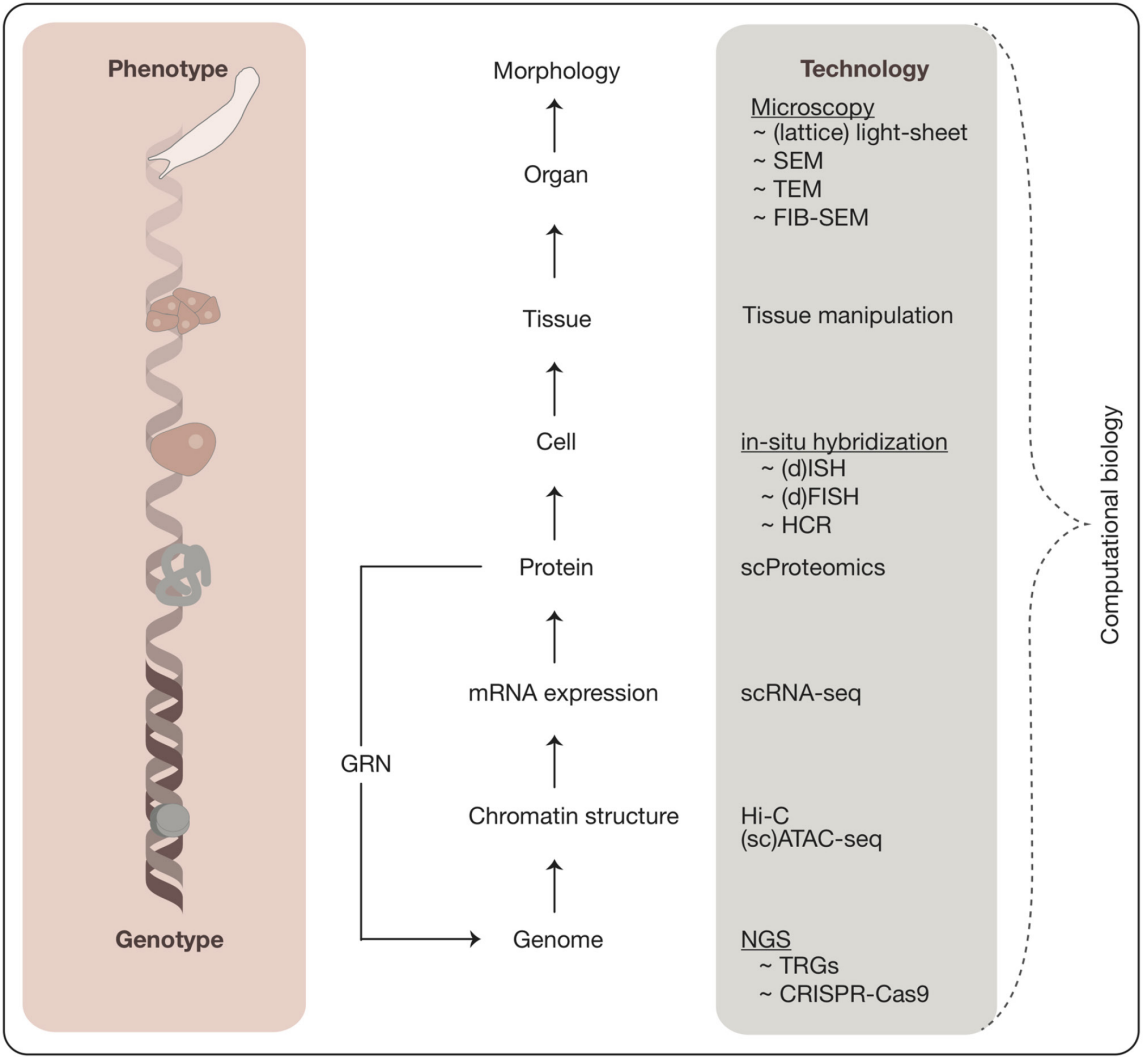
Bridging the genotype–phenotype gap. Highlighted techniques (grey) contribute to a detailed understanding of gene regulation on the different levels of the central dogma and enable causal connection of genotypes to phenotypes (pink). Abbreviations: CRISPR‐Cas9, clustered regularly interspaced short palindromic repeats; dFISH, double fluorescent in‐situ hybridization; FIB‐SEM, focussed ion beam scanning electron microscopy; HCR, hybridization chain reaction; ISH, in‐situ hybridization; NGS, next generation sequencing; scATAC‐seq, single‐cell assay for transposase‐accessible chromatin sequencing; scProteomics, single‐cell proteomics; scRNA‐seq, single‐cell RNA sequencing; SEM, Scanning electron microscopy; TEM, transmission electron microscopy; TRGs, taxonomically restricted genes.

**FIGURE 2:**
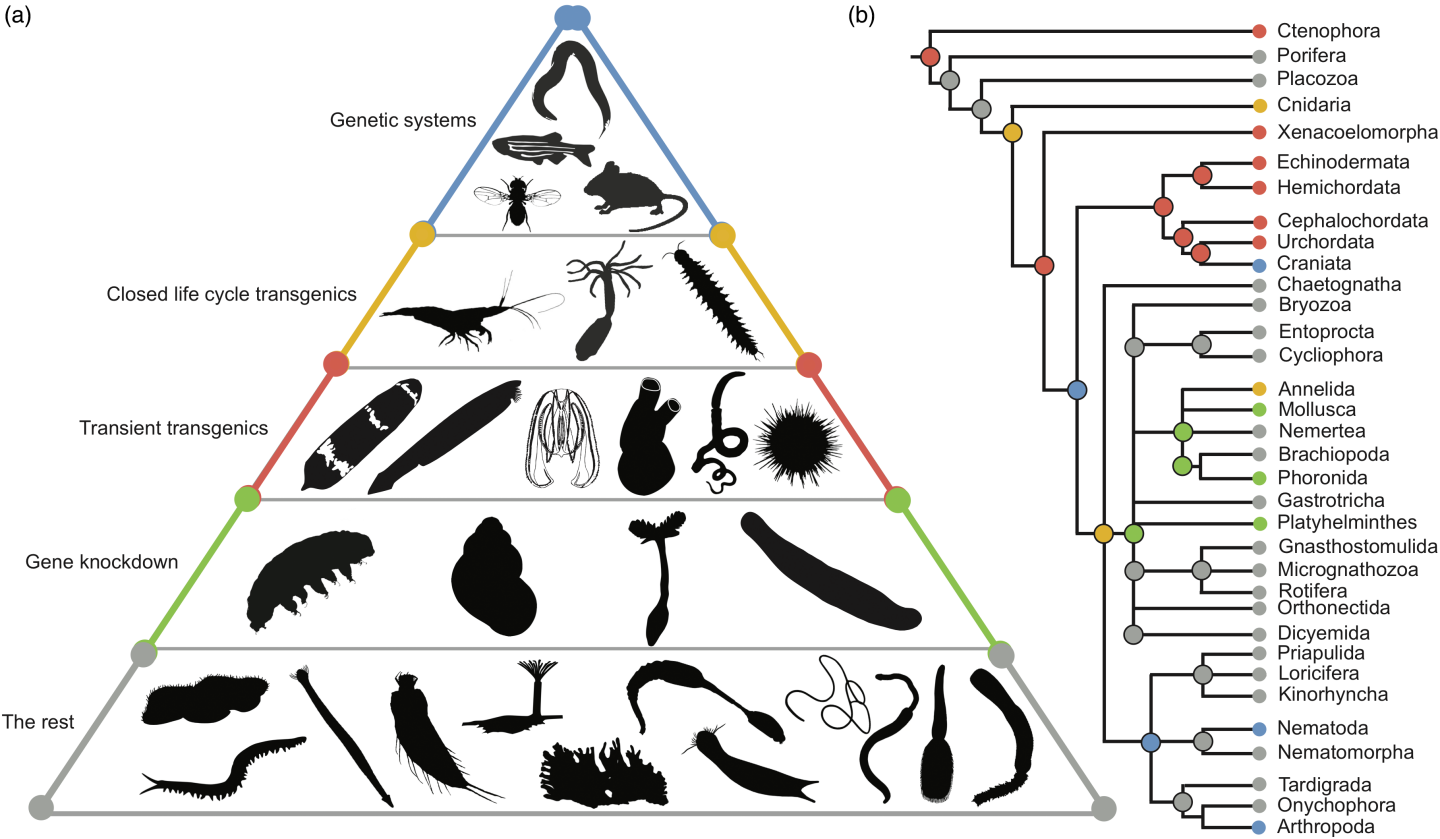
Pyramid of animal research organisms. Schematic representation of selected animal model systems (a) and their phylogeny (b) after (Dunn et al., 2014). Animals are separated into different categories from genetic systems (blue), over closed life cycle transgenics (yellow), transient transgenics (red), and gene knockdown (green) to all others (grey). All images were freely accessible from PhyloPic, except *Macrostomum lignano* (Platyhelminth). The Kinorhyncha (credit Noah Schlottman, photo by Martin V. Sørensen), Priapulida (credit Bruno C. Vellutini), Nematomorpha (credit Eduard Solà Vázquez, vectored by Yan Wong), Gastropoda (credit Armelle Ansart (photograph), Maxime Dahirel (digitization)), and Arthropoda (credit Maija Karala) silhouettes were used under the following license (https://creativecommons.org/licenses/by‐sa/3.0/) with no changes.

Illuminating the underlying molecular and cellular processes of how complex animals developed their unique morphology is the major aim of comparative developmental biology. Recent years led to the expansion of research on organisms that are studied regarding their development. This slowly overcomes previous limitations in understanding evolutionary developmental changes, which were mainly related to a lack of species (Love & Yoshida, 2019; Marx, 2021). Still, the number of completely decoded animal genomes is limited (Dunn & Ryan, 2015), which will likely change following global initiatives such as the Earth Biogenome Project (Lewin et al., 2022). However, the decoding of genomes is only describing the genotype of an organism and it is a long way to ultimately understanding the connection of genotype and phenotype, the genotype–phenotype map. With the study of the development of species, we are able to understand this connection which spans multiple levels of biological organization, from gene regulation through chromatin changes, and regulatory networks to cellular communication and cellular physical changes that ultimately lead to the specification of shape (Figure 1). While these connections become clearer in classical model systems, such as *Caenorhabditis elegans* and *Drosophila melanogaster*, the limited studies in other species leave a blind spot in our understanding of the evolution of genotype–phenotype connections. As a result, a large amount of biased information about traditional model organisms exists, but in contrast, very little is available about other species (Figure 2a). Currently, only around 30 species are classified as “model organisms,” whereas 8.7 million eukaryotic species exist globally of which 91% of marine species are yet to be discovered (Mora et al., 2011). Only 20–30% of marine animals have been discovered, although they represent up to 1.4 million animals worldwide (Costello et al., 2010). The study of additional species relies on their accessibility, which is often hampered by their ecology and life cycles. Overcoming this challenge is key to achieving an understanding of the evolution of animal diversity. The goal is to increase taxon sampling along all major lineages of the animal tree of life. Such an expansion leads to the reconstruction of character states at several nodes in the animal tree and by connecting these dots, the understanding of evolutionary changes of developmental mechanisms that led to animal diversity.

### 1.2 Evolutionary developmental biology is driven by advances in technology

Often declared as the emergence of a new field of research, already the birth of the area which we call shortly “evo‐devo” was initiated by the implementation of molecular methods into the field of comparative developmental biology, which existed long before (Hall, 2012). In the last decades, novel genomic, molecular, and microscopic technologies now allow connecting different levels of biological organization, such as 3‐ or 4‐dimensional regulatory genomic changes to cellular transcriptomics, proteomics, and ultrastructure. This is a steppingstone in understanding genotype to phenotype connections and provides the tools to understand homology on different organismic levels. Combining wet laboratory science and computational technologies enables the resolution of phylogenies, therefore increasing the number of accurate phylogenetic interpretations and the molecular detail to which these changes are identifiable. For example, combining transmission electron microscopy (TEM) with gene expression data in correlative microscopy provides information about the molecular as well as cellular localization of a gene of interest. Multi‐omics approaches, including, but not limited to proteome, epigenome, metabolome, and microbiome, will complement these datasets. We now have the technologies at hand to trace minute changes in cell–cell interactions, and thus the ability to define how different life forms emerged. Here, we highlight some of the recent most impactful technological advances and describe how these have progressed and shaped the direction and current state‐of‐the‐art in evo‐devo.

#### 1.2.1 Genomic and transcriptomic data

The increased accessibility and cost‐effectiveness of sequencing techniques enable whole genome sequencing projects with chromosome resolution across a broad range of species (Hobert, 2010). Sampling more species across a range of clades has and will produce genomic data which allows understanding evolutionary changes on the genomic level much better. Thus, properties, such as gene content, micro‐, and macrosyntenies, and chromosomal rearrangements in the genomic organization allow the reconstruction of important phylogenetic nodes (Simakov et al., 2022). The availability of more genomes enables the identification of more novel genes, in particular taxonomically restricted genes which are only present in certain clades or species (Johnson, 2018). The growing amount of additional genomes across animals and the development of novel algorithms support better orthologue detection (Martín‐Durán et al., 2017). User‐friendly software supporting orthologue assessments have been developed (Emms & Kelly, 2019). Next‐generation sequencing (NGS) techniques, reviewed elsewhere (da Fonseca et al., 2016), resolved many of the technical issues, including its affordability. At the same time, labourious genomic methods such as real‐time qPCR and micro‐arrays have now been replaced by single‐cell genomics/transcriptomics (Marioni & Arendt, 2017). Sequencing the entire nucleic acid content of organisms has become standardized and rapid. However, while advanced sequencing technologies, such as single‐cell omics provide novel information, the evaluation of previous datasets on the organismic level becomes even more crucial but is also more difficult and labour intensive.

#### 1.2.2 In‐situ hybridization assays

From its first establishment (Gall & Pardue, 1969), to the use of non‐radioactive colorimetric (Tautz & Pfeifle, 1989) and fluorescent probes (Langer‐Safer et al., 1982), in‐situ hybridization (ISH) has always been a key method in studying evolutionary developmental biology. The use of multiple fluorescent probes circumnavigated the labour and amount of tissue required for standard single gene ISH, as well as enabled concurrent visualization of the co‐expression of multiple genes (Rudkin & Stollar, 1977; Speicher & Carter, 2005). The recent development of the hybridization chain reaction (HCR) uses small, self‐assembling DNA monomers that are able to trigger customized, aptamer‐directed nucleic acid binding (Dirks & Pierce, 2004). The HCR principle using these metastable hairpins that hybridize to the target sequence upon initiator exposure (Dirks & Pierce, 2004) is superior in multiplexing, sample penetration, temperature, and signal‐to‐noise ratio (Choi et al., 2010, 2014, 2018). This allows to gain a higher resolution of the co‐expression of multiple genes in even small organisms. Recently, HCR was modified to label antibodies, which allows a higher resolution in immunohistochemistry (HCR IHC; Schwarzkopf et al., 2021).

#### 1.2.3 Microscopy and live imaging techniques

Traditional microscopic techniques (bright‐field, differential interference contrast, and fluorescent microscopy) are key to visualizing an organism's morphology, such as during ontogeny and performing comparative morphological analyses across extant species, as well as localization of gene expression patterns. Considering the spatiotemporal restrictions, it is now becoming possible to image entire life stages using light‐sheet microscopy, depending on their overall size, in several dimensions with a little background, photo‐bleaching, and sample damage compared to traditional confocal microscopy (Wan et al., 2019). The relatively new advanced imaging technique lattice light‐sheet has been successfully used to, among other biological processes, characterize protein interactions during embryogenesis in two of the six predominant model organisms, *C. elegans* and *D. melanogaster* (Chen et al., 2014). Since the technique is superior to traditional fluorescent imaging in speed, resolution, and resilience it is likely going to revolutionize evolutionary developmental biology further. Advances in computational image processing and machine learning improve automated cell lineage reconstruction (Hu et al., 2021; Lugagne et al., 2020). Electron microscopy, scanning (SEM) and TEM, have been used for many years to image biological specimens in 3‐ and 2D with a more than 800‐fold higher resolution compared to standard light microscopy. However, the focussed‐ion‐beam SEM (FIB‐SEM) is superior in *z*‐axis resolution, and has been used to characterize the brain connectome of *D. melanogaster* in 3D, the first complete map of its kind (Scheffer & Meinertzhagen, 2019; Scheffer et al., 2020; Xu et al., 2017). Also here, image‐processing algorithms and machine learning continuously improve the reconstructions and provide reliable and detailed datasets on the ultrastructural level (Heinrich et al., 2021; Xiao et al., 2018).

#### 1.2.4 Gene knockout/down and genome editing

Gene knock‐downs and knock‐outs are used in developmental biology to study the roles of genes during organismal development. The zebrafish spearheaded the development of reverse genetics using less invasive tools such as morpholinos and RNA inference (Carpio & Estrada, 2006). More recently, clustered regularly interspaced short palindromic repeats (CRISPR‐Cas9 (Jinek et al., 2012)) for gene knock‐down by genome editing have been established in marine invertebrates, for example, the tunicate *Ciona intestinalis* (Gandhi et al., 2018; Sasaki et al., 2014; Stolfi et al., 2014) and the ctenophore *Mnemiopsis leidyi* (Presnell & Browne, 2021). Other applications and species include, but are not limited to: the nervous system of Cnidaria (Ikmi et al., 2014), the dorsoventral patterning in Echinoidea (Lin & Su, 2016), and β‐catenin expression in Mollusca (Perry & Henry, 2015). However, to go beyond the studies of only the first function of these genes during embryogenesis, it is necessary to develop conditional knock‐outs with CRISPR‐Cas9, that allow the study of the function of the same gene in later developmental stages. These first applications in more commonly used systems pave the road for the implementation of CRISPR‐Cas9 in other species (Shen et al., 2014).

#### 1.2.5 Bioinformatics

Despite the recent popularity of bioinformatics as a cause of implementation of NGS technologies, the internet, and affordable, powerful machines, the field is more than 50 years old (Gauthier et al., 2019). In correlation with decreasing cost and advancement of NGS, the volume and complexity of sequencing and microscopic data have increased significantly over the past decade. Computational capabilities required to process, analyse, and store these generated large‐scale datasets have thus adapted as well (Schadt et al., 2010). In addition, the development of new algorithms, the implementation of machine learning and artificial intelligence drive all fields of research (Feltes et al., 2018). Its progress is closely tied to the advances in other techniques such as in principle all methods listed above.

### 1.3 Choosing the research organism

Despite technical advances in the methodologies, they come with financial and personnel costs. Since resources are often limited, the selection of suitable research species is crucial. Some technical considerations include accessibility/availability, cultivability, size, phylogenetic position, standardization, cost, and techniques. Different strategies for selecting research organisms have been proposed in the past. Some claim that evolutionary developmental biology requires a systematic and strategic approach to selecting species based on their ability to provide insight into a specific hypothesis, instead of a random selection (Jenner & Wills, 2007). Others support a focus on a few, closely related species to resolve exemplified mechanisms of evolutionary change and make use of the easiness to establish novel sophisticated methods (Sommer, 2009). During the last decade, both approaches have been followed in parallel and both have led to fundamental insights in the field. More recently, 20 empirical and philosophical criteria, applicable to essentially any research area, have been established aiding to identify, refine, and select suitable research organisms (Dietrich et al., 2020).

### 1.4 Advantages of marine organisms as model systems and the importance of marine evo‐devo

Since animals have developed from primary life forms originating in the ocean (Canfield et al., 2007), many marine animals inherently possess traits and morphological features that date back to the origins of arguably all essential animal organ systems: nervous system, excretory organs, musculature, digestive systems, and reproductive organs. Therefore, the importance of choosing marine species, in the context of evo‐devo and in relation to each individual research question, becomes evident. The knowledge gained allows the understanding of the evolution of non‐marine animals and their elaboration and diversification of the organ systems. Studying marine species enables researchers to draw conclusions about the characteristics of the last common ancestors at deep phylogenetic nodes (Figure 2b). Basic research on marine animals furthermore led to the discoveries of new cellular and physiological mechanisms, as is also highlighted by the range of Nobel prizes awarded (Table 1). The establishment of scalable lab‐based rearing and aquaculture protocols has further increased the usability of marine animals, such as for example *Nematostella vectensis* (Stefanik et al., 2013), *Platyneris dumerilii* (Kuehn et al., 2019), and *Ciona intestinalis* (Joly et al., 2007). For other species, automated aquaculture systems have improved the husbandry of marine invertebrates significantly (Henry et al., 2020). Despite the obvious phylogenetic and morphological insights marine animals can provide, they also pose limitations. Biology relies on the capacity for collection or culture of specimens, which implies that animals requiring highly specialized environmental conditions may be unculturable. To investigate different life stages, it is therefore crucial to monitor seasonal changes, reproductive seasons, and spawning patterns (Arnone & Hejnol, 2015). Their minimal use in the past stems primarily from technical limitations, for example, the inability to collect sufficient tissue due to their small body size (millimetre to centimetre range). The high‐risk, high‐gain component of these types of endeavours is essential, when if done successfully the research yields novel insights that could never be gained by studying traditional model systems.

**TABLE 1:** Award‐winning marine organisms.

Species		Award year	Discipline	Topic	Awardees	References
*Aequorae victoria*	GFP 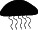	2008	Chemistry	Discovery and development of the green fluorescent protein, GFP	Osamu Shimomura Martin Chalfie Roger Y. Tsien	Chalfie et al. (1994); Heim (1995); Roda (2010); Shimomura et al. (1962)
*Arbacia punctulata*	Cyclin 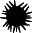	2001	Medicine	Discoveries of key regulators of the cell cycle	Leland H. Hartwell Sir Richard Timothy Hunt Sir Paul M. Nurse	Evans et al. (1983)
*Aplysia californica*	Memory 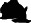	2000	Medicine	Discoveries in signal transduction in the nervous system	Arvid Carlsson Paul Greengard Erik R. Kandel	Carlsson et al. (1958); Greengard (2000); Johnson et al. (1971)
*Loligo pealeii*	Nerve cell excitation 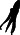	1963	Medicine	Ionic mechanisms involved in excitation and inhibition in the peripheral and central portions of the nerve cell membrane	Sir John Carew Eccles Alan Llyod Hodgkin Andrew Fielding Huxley	Hodgkin and Huxley (1952a, 1952b, 1952c); Hodgkin et al. (1952)
*Actinia sulcata Physalia physalis*	Anaphylaxis 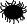 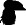	1913	Medicine	In recognition of his work on anaphylaxis	Charles Robert Richet	Richet and Portier (1902)
*Luidia sarsi*	Immunity 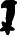	1908	Medicine	In recognition of their work on immunity	Ilya Ilyich Mechnikov Paul Ehrlich	–

*Note*: Collection of marine organisms which were the research subjects of topics that have been awarded a Nobel prize (https://www.nobelprize.org/prizes/, accessed 7th Aug 2021; https://fbresearch.org/medical‐advances/nobel‐prizes/, accessed 7 August 2021).

## 2 AN OUTLOOK FOR MARINE EVOLUTIONARY DEVELOPMENTAL BIOLOGY

The rapid technological progress allows the implementation of these technologies into more species including species from the marine environment. The next steps forward in the field will be made by combining these methods. This will allow us to bridge and co‐investigate different levels of biological organization simultaneously (Figure 1). Single‐cell omics will be combined with ultrastructural investigations to draw a coherent description of cell states during embryogenesis. Micromanipulations and laser surgery studies will add the cellular behaviour and cell shape changes of these cells. It seems, that through these developments, the cells regain importance in the focus of evolutionary developmental biology as the most basic and smallest living unit of the embryo, that ultimately determines its shape. The phylogenetic position of many marine organisms makes them indispensable to answer some of the most important unresolved questions: Which came first, Porifera or Ctenophora? What are the phylogenetic relationships within the Spiralia? The coming years will lead to a more complete picture of the genotype–phenotype relationship and ultimately guide us to understand the evolution of development.

## CONFLICT OF INTEREST

The authors declare no competing interest.

## Data Availability

Data sharing is not applicable to this article as no new data were created or analyzed in this study.
